# Antiseptic Treatment in Extraction of Cataract

**Published:** 1879-09

**Authors:** 


### ANTISEPTIC TREATMENT IN EXTRACTION OF CATARACT.

Professor Alfred Graefe, of Halle, has become a convert to the antiseptic treatment in certain cases, and from his experience one is not surprised to see him preaching the propaganda concerning it. Out of one hundred and fourteen operations, for extraction of cataract, he reports an entire loss of only three cases—a result which certainly commends the method to careful consideration.

Moreover, the plan which he adopts is by no means as complicated as Lister's multitudinous bandages. On the day before the operation, besides applying atropine, he washes the entire conjunctiva with a watery solution of carbolic acid, about nine grains to the ounce, (2 per cent.,) and then closing the eye, covers it with a sponge dipped in the same fluid. He is careful that all instruments and other articles coming in contact with the eye are washed in a similar solution.

It is also used for cleansing the globe during and after the operation, and finally a piece of lint moistened in a 4 per cent. solution of boric acid, (xviii grains to the ounce,) having been laid on the lids, the bandage is applied. At each subsequent dressing the same solutions are also used for bathing and dressing the eye, until the wound is firmly closed, and the process of healing far advanced.

The plan is certainly a simple one, and being recommended by so careful and conscientious an observer, will find many to adopt it.

